# Clinical Impact of the BIOFIRE® FILMARRAY® Pneumonia Panel in the Veterans Affairs Healthcare System: A Difference-in-Differences Study

**DOI:** 10.1093/cid/ciag328

**Published:** 2026-06-12

**Authors:** Nicholas S Britt, Karim Khader, Tao He, Andrea M Prinzi, Benjamin Hommel, Tristan T Timbrook, Emily M Potter, Thomas P Lodise

**Affiliations:** Department of Pharmacy Practice, University of Kansas School of Pharmacy, Lawrence, Kansas, USA; Department of Internal Medicine, University of Kansas School of Medicine, Kansas City, Kansas, USA; Pharmacy Service, Dwight D. Eisenhower Veterans Affairs Medical Center, Leavenworth, Kansas, USA; Department of Internal Medicine, University of Utah School of Medicine, Salt Lake City, Utah, USA; IDEAS Center of Innovation, Veterans Affairs Salt Lake City Health Care System, Salt Lake City, Utah, USA; Department of Internal Medicine, University of Utah School of Medicine, Salt Lake City, Utah, USA; IDEAS Center of Innovation, Veterans Affairs Salt Lake City Health Care System, Salt Lake City, Utah, USA; bioMérieux, Marcy L’etoile, France; bioMérieux, Marcy L’etoile, France; Pharmacy Department, Barnes-Jewish Hospital, St. Louis, Missouri, USA; Pharmacy Service, Dwight D. Eisenhower Veterans Affairs Medical Center, Leavenworth, Kansas, USA; Department of Pharmacy Practice, Albany College of Pharmacy and Health Sciences, Albany, New York, USA; Pharmacy Serivce, Samuel S. Stratton Veterans Affairs Medical Center, Albany, New York, USA

**Keywords:** pneumonia, rapid diagnostic testing, BIOFIRE, pneumonia panel antimicrobial stewardship, Veterans Health Administration

## Abstract

**Background:**

Rapid diagnostics such as the BIOFIRE® FILMARRAY® Pneumonia (PN) Panel may enhance antibiotic optimization, yet real-world evidence remains limited, and few studies have assessed how differences in institutional antimicrobial stewardship (ASP) capacity influence clinical outcomes.

**Methods:**

We conducted a national, multicenter retrospective study of ICU patients with pneumonia admitted to Veterans Health Administration hospitals. Hospitals that implemented the PN Panel for ≥6 months served as intervention sites, while matched facilities without implementation served as controls. Outcomes were assessed using a difference-in-differences framework with inverse probability weighting to account for baseline imbalances and secular trends, including those related to the COVID-19 pandemic. Co-primary outcomes, assessed following index culture collection, were early antimicrobial de-escalation (24–48 hours) and early appropriate therapy (0–24 hours); secondary outcomes were 30-day and ICU mortality.

**Results:**

The study included 11 746 patients, including 4523 from hospitals that implemented the BIOFIRE® PN Panel and 7223 from matched control hospitals. Overall, PN implementation was associated with early antimicrobial de-escalation (adjusted RR [aRR], 1.14; 95% CI, 1.04–1.26). Among culture-positive patients, PN Panel implementation was significantly associated with early antimicrobial de-escalation (aRR, 1.23; 95% CI, 1.02–1.50) and early appropriate therapy (aRR, 1.11; 95% CI, 1.04–1.18). Overall ICU and 30-day mortality did not differ, though ICU mortality among culture-positive patients was relatively lower following PN Panel implementation (aRR, .70; 95% CI, 0.50–0.97). Improvements in antimicrobial optimization were most evident in hospitals with greater ASP capacity.

**Conclusions:**

BIOFIRE® PN Panel implementation improved antibiotic optimization in critically ill pneumonia patients, with the greatest gains seen in hospitals with comprehensive ASP programs.

## Teaser

In this national, multicenter retrospective study of intensive care unit patients with pneumonia in Veterans Health Administration hospitals, BIOFIRE® PN Panel implementation was associated with improved early antimicrobial de-escalation and appropriate therapy, with the greatest benefit in hospitals with comprehensive antimicrobial stewardship infrastructure.

**(See the Editorial Commentary by Klompas and Rhee on pages e472–4.)**

Pneumonia is a leading cause of morbidity and mortality among hospitalized patients and one of the most common infections managed in intensive care units (ICUs) [1–3]. Rapid diagnostic tests (RDTs) may improve pneumonia management in ICU patients by expediting appropriate therapy for multidrug-resistant (MDR) infections and enabling earlier antimicrobial de-escalation [4–9]. The BIOFIRE® FILMARRAY® Pneumonia (PN) Panel (BioFire Diagnostics, LLC, Salt Lake City, Utah, USA) is a Food and Drug Administration–cleared RDT that uses multiplex polymerase chain reaction (PCR) to detect 18 bacterial targets, 8 viral targets, and 7 antimicrobial resistance genes from respiratory specimens. Prior studies suggest RDTs are most effective when integrated with antimicrobial stewardship programs (ASPs), improving time to therapy, mortality, and length of stay, although evidence in critically ill pneumonia patients remains limited [10, 11].

The Veterans Health Administration (VHA), the largest integrated healthcare system in the United States, provides care to nearly 6 million enrollees and manages more than 700 000 inpatient admissions annually across 130 medical centers [12]. Although ASPs are required across VHA hospitals, stewardship practices vary locally, creating an opportunity to evaluate the impact of RDT implementation across different stewardship environments. This study evaluated the real-world clinical impact of BIOFIRE® PN Panel implementation among critically ill patients with pneumonia treated within the VHA system overall and across institutions with differing ASP practices.

## METHODS

### Study Design and Population

This was a national, multicenter retrospective quasi-experimental study of ICU patients with pneumonia admitted to inpatient VHA hospitals between 1 January 2016 and 31 December 2024. Hospitals were classified as intervention sites if they implemented the BIOFIRE® PN Panel for ≥6 months during the study period, and as control sites if they did not implement the Panel. Intervention hospitals were matched 1:1 to control hospitals based on facility complexity level and geographic region prior to cohort assembly. In total, 36 hospitals contributed patients to the analysis, including 18 intervention hospitals and 18 matched control hospitals.

To estimate the effect of PN Panel implementation, we applied a difference-in-differences (DiD) design, comparing pre- and postimplementation outcomes at intervention sites (PN Panel group) against contemporaneous changes at control sites. This approach allowed estimation of the effect of BIOFIRE® PN Panel implementation by comparing changes over time at intervention hospitals with concurrent changes at matched control hospitals, thereby accounting for secular trends in healthcare delivery, including those associated with the COVID-19 pandemic [13, 14].

Pneumonia cases were identified and data collected during the period 3 years before and 3 years after PN Panel implementation, excluding a 2-month wash-in period. For each intervention hospital, the study timeline therefore consisted of 3 periods relative to the PN Panel implementation date: a 3-year preimplementation period, a 2-month wash-in period (excluded from analysis), and a 3-year postimplementation period; corresponding calendar periods were applied to matched control hospitals.

Eligible patients in the PN Panel group met the following criteria: (1) inpatient admission to a facility utilizing the PN Panel; (2) clinical evidence of pneumonia, defined by natural language processing of radiology reports indicating new, progressive, or persistent lung infiltrate, consolidation, or cavitation [15]; (3) receipt of ≥1 antimicrobial agent within 48 hours of pneumonia diagnosis; (4) ICU residence at the time of pneumonia diagnosis or ICU admission within 24 hours of diagnosis; (5) respiratory culture (sputum, bronchoalveolar lavage [BAL], or tracheal aspirate) obtained within 48 hours of pneumonia diagnosis; (6) PN Panel performed on the same specimen as the index respiratory culture (postimplementation group only); and (7) survival >24 hours after index respiratory specimen collection. Clinical pneumonia diagnosis was used to identify the eligible pneumonia episode, whereas index respiratory specimen collection was used as the analytic time zero for microbiology-informed treatment outcomes.

In the postimplementation period, restricting the cohort to cases in which the PN Panel was performed on the same respiratory specimen as the index culture ensured that the diagnostic exposure reflected patients whose clinical management could plausibly be influenced by the rapid diagnostic result. If a patient had >1 pneumonia episode during the study period, only the first qualifying episode was analyzed. The parallel control (non-PN Panel) group included patients who met the same eligibility criteria at hospitals that had not implemented the PN Panel during the same calendar periods corresponding to the pre- and postimplementation windows of their matched intervention hospital [16].

### Data Collection

Pneumonia cases were identified through a query of the VHA Corporate Data Warehouse, a national patient-level clinical and administrative database of all VHA outpatient and inpatient encounters. Variables collected included patient demographics, admissions, comorbidities [17], disease severity (measured by the modified APACHE-II score [18]), antimicrobial administration, microbiology, laboratory, and mortality data. Data regarding ASP programs were obtained from the 2020 Healthcare Analytics and Information Group (HAIG) survey of VHA facilities (N.S.B. data use agreement) [19]. This survey captured information on stewardship program structure and policies at the facility level. Survey responses were used to classify hospitals according to the presence or absence of specific stewardship components (eg, infectious diseases consult services, antibiotic timeout policies, automatic antibiotic stop orders, MRSA nasal screening protocols, and disease-specific treatment pathways). These variables were treated as binary facility-level indicators and linked to all patient encounters occurring at the corresponding hospital. This study was approved by institutional review boards at Albany, Kansas City, and Salt Lake City VHA medical centers.

### Microbiology

All respiratory specimens underwent standard culture and susceptibility testing in local VHA microbiology laboratories according to Clinical and Laboratory Standards Institute (CLSI) guidelines current at the time of culture collection [20]. Bacterial targets included in the BIOFIRE® PN Panel were classified as “Panel” organisms, and those not included as “non-Panel” organisms. Concordance between PN Panel and culture results was assessed in the postimplementation group, with positive and negative percent agreement (PPA/NPA) calculated for each target organism using standard culture as the reference standard. Resistance determinants were identified by PN Panel results and resistance gene targets, when reported [21].

### Clinical Outcomes

Co-primary outcomes, assessed following index culture collection, were early antimicrobial de-escalation (24–48 hours after index culture collection) and receipt of early appropriate therapy (0–24 hours after index culture collection). Early antimicrobial de-escalation was defined as any reduction in the total Antibiotic Spectrum Coverage (ASC) score from 0–24 to 24–48 hours after index culture collection and was assessed overall and among the subgroup of patients with respiratory culture positive for a BIOFIRE® PN Panel bacterial pathogen [22, 23]. ASC scores were calculated based on antimicrobial agents administered during each time interval. If a patient died during the 24–48 hour window, only antibiotics administered prior to death contributed to the ASC calculation. Early appropriate therapy was evaluated only among culture-positive patients because appropriateness cannot be determined without identification of a causative pathogen. Appropriate therapy was defined as administration of ≥1 dose of an antimicrobial agent to which the corresponding organism was susceptible, or inferred to be susceptible based on CLSI recommendations [20]. In cases of polymicrobial lung infection, appropriate therapy was required for all organisms identified from the respiratory specimen. Secondary outcomes included ICU mortality, 30-day all-cause mortality, 90-day hospital readmission, 60-day incidence of MDR infection, and 90-day incidence of *Clostridioides difficile* infection (CDI).

### Statistical Analysis

Initial unadjusted descriptive statistics were performed to characterize patients pre- and postimplementation for the intervention and control groups. Baseline categorical variables were compared by χ^2^ test or 2-tailed Fisher's exact test, and continuous variables were compared by *t*-test or Wilcoxon rank-sum method. To ensure balance in baseline characteristics between groups, inverse probability of treatment weighting (IPTW) was applied using a propensity score model. The propensity score estimated the probability of exposure to PN Panel implementation based on baseline patient and clinical characteristics. Propensity scores were estimated using logistic regression and included age, sex, comorbidities, pneumonia type (community-acquired vs hospital-acquired), mechanical ventilation, respiratory sample type, modified APACHE-II score [24], prior hospitalization, prior COVID-19 infection, systemic inflammatory response syndrome (SIRS) criteria, sepsis (defined as ≥2 SIRS criteria or ICD-10 code), and immunosuppression. The resulting weights were applied to balance measured covariates between comparison groups. All models included month of index pneumonia diagnosis as a covariate to control for temporal trends and seasonality. Extreme weights from the propensity score model were truncated at the 1st and 99th percentiles. Baseline balance after IPTW was assessed using standardized mean differences (SMDs), with an SMD <0.1 indicative of adequate balance.

The effect of PN Panel implementation was evaluated using generalized linear models with a Poisson distribution and robust variance estimates. These models included fixed effects for hospital and time, along with an interaction term for intervention status (PN Panel vs control) and implementation period (pre vs post). The parallel trends assumption was assessed both visually, by plotting preintervention trends in outcomes, and statistically by fitting weighted regression models in the preintervention period including an interaction term between event time and treatment group to test for differences in outcome trajectories over time. The effect of PN Panel implementation on the co-primary outcomes and 30-day mortality were further evaluated in a series of DiD Poisson regression analyses stratified by varying ASP practices [19] and pneumonia type (hospital- vs nonhospital-acquired), with effect modification assessed using a 3-way interaction term. All subgroup analyses were determined a priori and considered hypothesis-generating, without adjustment for multiple comparisons. Statistical analyses were performed using R (version 4.4.2, R Foundation for Statistical Computing, Vienna, Austria) and a 2-tailed *P* value < .05 was considered statistically significant for all tests [25].

## RESULTS

Overall, 11 746 patients met study criteria, including 4523 from hospitals that implemented the BIOFIRE® PN Panel (3550 preimplementation and 973 postimplementation) and 7223 from matched control hospitals (3671 pre-implementation and 3552 postimplementation; Supplementary Figure 1). A total of 36 hospitals contributed patients to the analysis, including 18 hospitals that implemented the BIOFIRE® PN Panel and 18 matched control hospitals. Baseline characteristics were compared between pre- and postimplementation periods for both groups, with several statistically significant differences observed (Table 1). Postimplementation patients were older and more likely to be mechanically ventilated, have hospital-acquired pneumonia, undergo BAL sampling, and present with sepsis. Prior COVID-19 infection was more common in the postimplementation period, consistent with temporal trends. Within each implementation period, baseline characteristics were generally comparable between PN and non-PN hospitals.

**Table 1.** Baseline Characteristics of Critically Ill Pneumonia Patients Across PN Panel and Control Hospitals in the Pre- and Postimplementation Periods

	PN Panel Hospitals			Control Hospitals		
Characteristic (N = 11 746)	Preimplementation period (n = 3550)	Postimplementation period (n = 973)	*P*	Preimplementation period (n = 3671)	Postimplementation period (n = 3552)	*P*
Age (years), median (Q1, Q3)	71 (64, 76)	72 (65, 77)	.002	71 (65, 76)	73 (66, 77)	<.001
Age ≥65 y, n (%)	2661 (75.0)	748 (76.9)	.219	2769 (75.4)	2770 (78.0)	.010
Male, n (%)	3443 (97.0)	935 (96.1)	.162	3542 (96.5)	3416 (96.2)	.477
Mechanically ventilated, n (%)	2442 (68.8)	717 (73.7)	.003	2591 (70.6)	2367 (66.6)	<.001
Time to infection from hospital admission (hours), median (Q1, Q3)	35 (9, 115)	55 (15, 174)	<.001	32 (8, 111)	44 (12, 142)	<.001
Hospital-acquired, n (%)^a^	1522 (42.9)	506 (52.0)	<.001	1554 (42.3)	1701 (47.9)	<.001
Year of pneumonia episode, n (%)	…	…	<.001	…	…	<.001
2016	32 (0.9)	0 (0.0)		34 (0.9)	0 (0.0)	…
2017	508 (14.3)	0 (0.0)		597 (16.3)	0 (0.0)	…
2018	950 (26.8)	0 (0.0)		850 (23.2)	0 (0.0)	…
2019	1006 (28.3)	2 (0.2)		976 (26.6)	11 (0.3)	…
2020	672 (18.9)	114 (11.7)		706 (19.2)	475 (13.4)	…
2021	241 (6.8)	196 (20.1)		393 (10.7)	806 (22.7)	…
2022	131 (3.7)	212 (21.8)		104 (2.8)	877 (24.7)	…
2023	10 (0.3)	286 (29.4)		11 (0.3)	906 (25.5)	…
2024	0 (0.0)	163 (16.8)		0 (0.0)	477 (13.4)	…
Specimen type, n (%)	…	…	<.001	…	…	<.001
Bronchoalveolar lavage	382 (10.8)	226 (23.2)		412 (11.2)	440 (12.4)	…
Sputum	3123 (88.0)	745 (76.6)		3249 (88.5)	3047 (85.8)	…
Tracheal aspirate	45 (1.3)	2 (0.2)		10 (0.3)	65 (1.8)	…
SIRS criteria, n (%)	…	…	.341	…	…	.007
0	663 (18.7)	172 (17.7)		672 (18.3)	582 (16.4)	…
1	1353 (38.1)	344 (35.4)		1388 (37.8)	1269 (35.7)	…
2	1074 (30.3)	317 (32.6)		1150 (31.3)	1191 (33.5)	…
3	413 (11.6)	125 (12.8)		410 (11.2)	464 (13.1)	…
4	47 (1.3)	15 (1.5)		51 (1.4)	46 (1.3)	…
Sepsis, n (%)	2323 (65.4)	712 (73.2)	<.001	2333 (63.6)	2455 (69.1)	<.001
Elevated lactate, n (%)	804 (22.6)	188 (19.3)	.026	790 (21.5)	760 (21.4)	.898
Acute kidney injury, n (%)	1820 (51.3)	525 (54.0)	.137	1818 (49.5)	1902 (53.5)	<.001
Vasopressor requirement, n (%)	1215 (34.2)	397 (40.8)	<.001	1088 (29.6)	1380 (38.9)	<.001
Inodilator requirement, n (%)	152 (4.3)	45 (4.6)	.642	116 (3.2)	121 (3.4)	.556
Hospitalization previous 90 d, n (%)	209 (5.9)	14 (1.4)	<.001	169 (4.6)	106 (3.0)	<.001
Prior COVID-19 infection, ever, n (%)	289 (8.1)	335 (34.4)	<.001	315 (8.6)	1177 (33.1)	<.001
Peptic ulcer disease, n (%)	171 (4.8)	41 (4.2)	.430	166 (4.5)	161 (4.5)	.983
Rheumatologic disease, n (%)	85 (2.4)	14 (1.4)	.071	90 (2.5)	76 (2.1)	.376
Chronic kidney disease, n (%)	2104 (59.3)	544 (55.9)	.060	2079 (56.6)	1883 (53.0)	.002
Peripheral vascular disease, n (%)	777 (21.9)	254 (26.1)	.005	876 (23.9)	810 (22.8)	.288
Hemiplegia/paraplegia, n (%)	246 (6.9)	67 (6.9)	.962	243 (6.6)	211 (5.9)	.234
Myocardial infarction, n (%)	618 (17.4)	159 (16.3)	.434	705 (19.2)	607 (17.1)	.020
Malignancy, any, n (%)	1024 (28.8)	290 (29.8)	.559	1016 (27.7)	1019 (28.7)	.339
Metastatic solid tumor, n (%)	319 (9.0)	93 (9.6)	.583	305 (8.3)	307 (8.6)	.610
Liver disease, mild, n (%)	589 (16.6)	137 (14.1)	.059	522 (14.2)	530 (14.9)	.398
Liver disease, moderate-severe, n (%)	373 (10.5)	71 (7.3)	.003	297 (8.1)	260 (7.3)	.220
Dementia, n (%)	430 (12.1)	100 (10.3)	.115	451 (12.3)	388 (10.9)	.071
Diabetes, uncomplicated, n (%)	1170 (33.0)	269 (27.6)	.002	1105 (30.1)	919 (25.9)	<.001
Diabetes, complicated, n (%)	982 (27.7)	257 (26.4)	.439	908 (24.7)	874 (24.6)	.899
Cerebrovascular disease, n (%)	683 (19.2)	188 (19.3)	.954	708 (19.3)	710 (20.0)	.452
Chronic obstructive pulmonary disease, n (%)	1971 (55.5)	590 (60.6)	.004	2291 (62.4)	2104 (59.2)	.006
Congestive heart failure, n (%)	1471 (41.4)	433 (44.5)	.086	1562 (42.5)	1491 (42.0)	.622
Human immunodeficiency virus infection, n (%)	43 (1.2)	8 (0.8)	.309	31 (0.8)	32 (0.9)	.796
Chronic hemodialysis dependence, n (%)	221 (6.2)	88 (9.0)	.002	222 (6.0)	238 (6.7)	.256
Charlson comorbidity index, median (Q1, Q3)	8.0 (5.0, 11.0)	8.0 (5.0, 11.0)	.221	8.0 (5.0, 11.0)	7.0 (5.0, 10.0)	.086
Modified APACHE II score, median (Q1, Q3)	13.0 (10.0, 16.0)	13.0 (10.0, 16.0)	.051	13.0 (10.0, 16.0)	13.0 (10.0, 16.0)	.644

Abbreviations: APACHE II, Acute Physiology and Chronic Health Evaluation II; SIRS, systemic inflammatory response syndrome.

^a^Pneumonia type (community-acquired vs hospital-acquired) was defined based on the timing of pneumonia diagnosis relative to hospital admission and administrative coding within the VHA Corporate Data Warehouse and was included as a covariate in the propensity score model.

### Microbiology

There were 3374 patients (28.7%) with a respiratory culture positive for at least 1 PN Panel organism, while 5991 patients (51.0%) had no organism identified; the remaining patients had cultures reported as normal respiratory flora or nonpanel organisms (Table 2). Gram-positive pathogens included on the BIOFIRE® PN Panel accounted for approximately 12%–14% of isolates, predominantly *Staphylococcus aureus*, with no significant differences pre- versus postimplementation. Gram-negative pathogens were more frequent after PN Panel implementation in intervention hospitals, with increases in *Pseudomonas aeruginosa*, *Proteus* spp., and *Klebsiella aerogenes*, while pathogen distributions remained stable in control hospitals.

**Table 2.** Respiratory Culture Results of Critically Ill Pneumonia Patients Across PN Panel and Control Hospitals in the Pre- and Postimplementation Periods

	PN Panel Hospitals (n = 4523)			Control Hospitals (n = 7223)		
Organism (N = 11 746)	Preimplementation Period (n = 3550)	Postimplementation Period (n = 973)	*P*	Preimplementation Period (n = 3671)	Postimplementation Period (n = 3552)	*P*
Panel organisms						
Gram-positive organism, n (%)	434 (12.1)	136 (14.0)	.145	516 (14.1)	477 (13.4)	.439
*S. aureus*	360 (10.1)	112 (11.5)	.216	423 (11.5)	406 (11.4)	.902
MRSA among *S. aureus*	158 (43.9)	53 (47.3)	.530	202 (47.8)	181 (44.6)	.362
*Streptococcus agalactiae*	18 (0.5)	3 (0.3)	.419	21 (0.6)	26 (0.7)	.465
*Streptococcus pneumoniae*	68 (1.9)	26 (2.7)	.162	88 (2.4)	55 (1.5)	.010
*Streptococcus pyogenes*	3 (0.1)	0 (0.0)	>.999	1 (0.0)	4 (0.1)	.211
Gram-negative *organism*	653 (18.3)	223 (22.9)	.002	672 (18.3)	660 (18.6)	.763
*A. baumannii complex*	17 (0.5)	5 (0.5)	.799	19 (0.5)	17 (0.5)	.814
*E. cloacae complex*	53 (1.5)	17 (1.7)	.569	57 (1.6)	48 (1.4)	.475
*Escherichia coli*	93 (2.6)	29 (3.0)	.538	114 (3.1)	83 (2.3)	.045
*Haemophilus influenzae*	100 (2.8)	24 (2.5)	.553	91 (2.5)	75 (2.1)	.298
*Klebsiella aerogenes*	27 (0.8)	15 (1.5)	.025	24 (0.7)	21 (1.2)	.024
*Klebsiella oxytoca*	22 (0.6)	9 (0.9)	.307	31 (0.8)	26 (0.7)	.589
*K. pneumoniae group*	113 (3.2)	39 (4.0)	.206	118 (3.2)	143 (4.0)	.065
*Moraxella catarrhalis*	24 (0.7)	10 (1.0)	.261	24 (0.7)	25 (0.7)	.796
*Proteus* spp.	41 (1.2)	23 (2.4)	.005	32 (0.9)	37 (1.0)	.470
*Pseudomonas aeruginosa*	219 (6.2)	91 (9.4)	<.001	230 (6.3)	206 (5.8)	.403
*Serratia marcescens*	39 (1.1)	13 (1.3)	.538	46 (1.3)	53 (1.5)	.382
Nonpanel organisms^a^						
Other Gram-positive	119 (3.4)	42 (4.3)	.150	142 (3.9)	167 (4.7)	.800
Other Gram-negative	151 (4.3)	59 (6.1)	.017	155 (4.2)	195 (5.5)	.012
Fungal	462 (13.0)	141 (14.5)	.230	505 (13.8)	577 (16.2)	.003
Normal flora	462 (13.0)	302 (31.0)	<.001	78 (2.1)	84 (2.4)	.491
No organism identified	1767 (49.8)	327 (33.6)	<.001	1995 (54.3)	1902 (53.5)	.497

^a^May include any of these alone or in addition to a Panel organism (ie, analysis not limited to Panel culture-positive cases only). Organism-specific categories are not mutually exclusive, as patients may have multiple organisms identified; however, “no organism identified” and “normal flora” represent mutually exclusive patient-level categories.

Among PN Panel-tested patients in the postimplementation period, a PN Panel-targeted bacterial pathogen was detected in 526 of 973 patients (54.1%), with a median time to result of 4.3 hours (IQR, 2.6–12.6). Concordance between PN Panel results and standard culture is shown in Supplementary Table 1. Overall performance demonstrated high positive percent agreement (PPA 95.2%) and negative percent agreement (NPA 96.5%). Antimicrobial resistance gene detection and atypical pathogens were uncommon (Supplementary Tables 2 and 3). Respiratory viruses were identified in 11.6% of specimens (113/973), and bacterial co-infections occurred in 7.3% of cases (71/973). Additional nonpanel bacterial pathogens identified on culture are summarized in Supplementary Table 4.

### Outcomes

In the overall unadjusted analyses, early antimicrobial escalation within 24–48 hours of index culture collection increased from 33.2% (n = 1179/3550) to 39.8% (n = 387/973) at PN Panel hospitals, compared with a smaller increase (33.8% [n = 1240/3671] to 35.6% [n = 1266/3552]) at control hospitals, yielding a DiD of 4.8% (risk ratio [RR], 1.13; 95% CI, 1.01–1.27; *P* = .027; Table 3, Figure 1, and Supplementary Table 5). The positive association between PN Panel implementation and early antimicrobial de-escalation persisted after IPTW adjustment in Poisson regression analysis (adjusted RR [aRR], 1.14; 95% CI, 1.04–1.26; *P* = .007; Figure 1 and Supplementary Table 5). Groups were well-balanced with regard to all measured covariates following IPTW adjustment (Supplementary Figure 2). There was no significant difference in median ASC score between postimplementation groups at the 24–48 hour time interval of antimicrobial treatment (PN panel, 12; interquartile range [IQR], 8–16 vs control, 12; [IQR, 8–16]; *P* = .154; Supplementary Figure 3).

**Figure 1.**
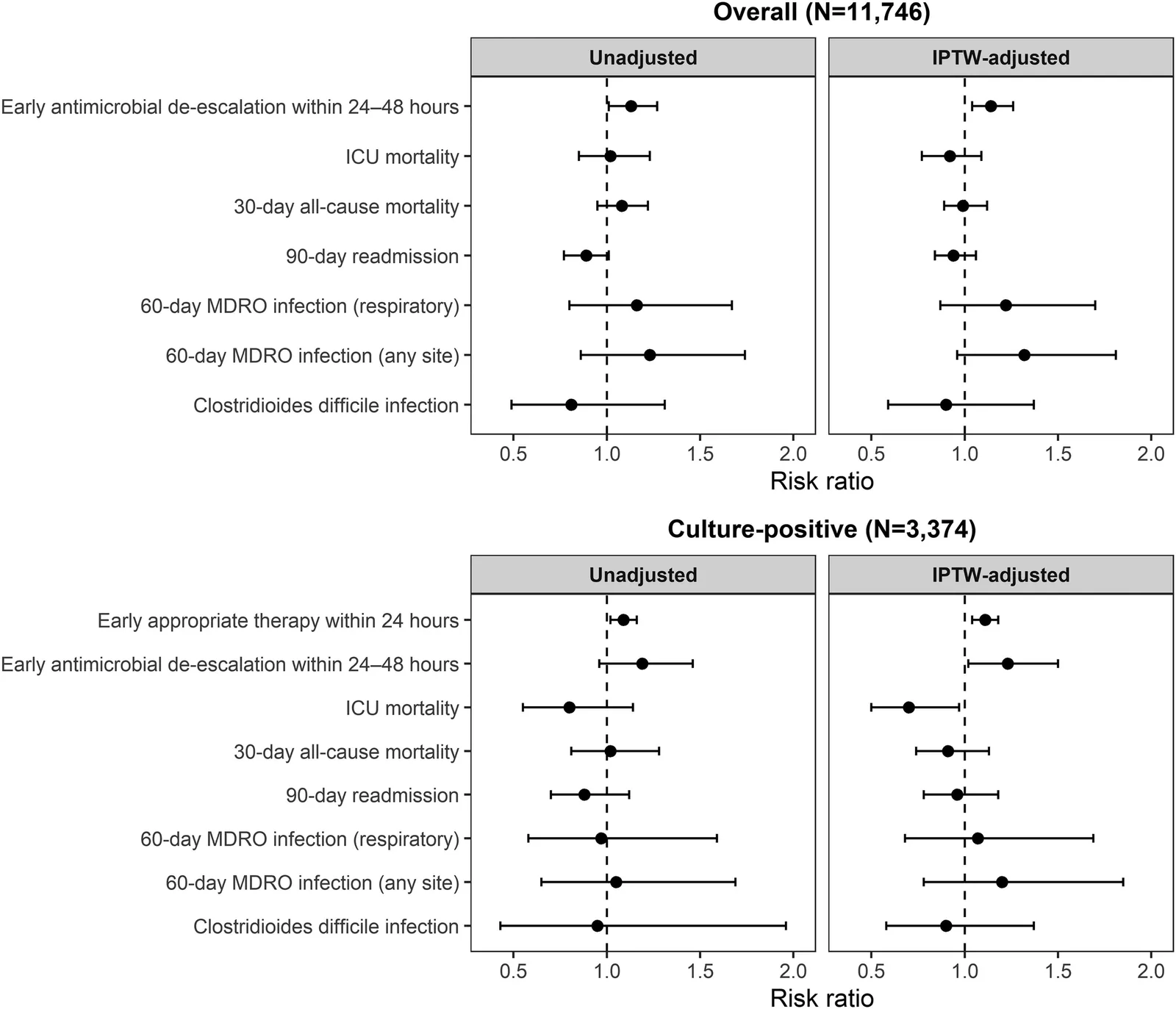
Difference-in-differences Poisson regression analysis of outcomes associated with PN Panel implementation. A risk ratio >1 indicates higher rates of the outcome associated with BIOFIRE(R) PN Panel implementation (post- vs preimplementation, relative to controls). Abbreviations: IPTW, inverse probability of treatment weighting; ICU, intensive care unit; MDRO, multidrug-resistant organism

**Table 3.** Unadjusted Differences in Clinical Outcomes Between Critically Ill Pneumonia Patients in PN Panel Hospitals and Control Hospitals

	PN Panel Hospitals			Control Hospitals			BIOFIRE-Control
Outcome	Preimplementation	Postimplementation	Diff	Preimplementation	Postimplementation	Diff	Diff-in-Diff
Overall (N = 11 746)							
Early antimicrobial de-escalation within 24–48 h, n (%)^a^	1179 (33.2)	387 (39.8)	6.6%	1240 (33.8)	1266 (35.6)	1.8%	4.8%
ICU mortality, n (%)	457 (12.9)	211 (21.7)	8.8%	425 (11.6)	680 (19.1)	7.5%	1.3%
30-d all-cause mortality, n (%)	963 (27.1)	348 (35.8)	8.7%	973 (26.5)	1151 (32.4)	5.9%	2.8%
90-d readmission, n (%)	1205 (33.9)	252 (26.9)	−7.0%	1191 (32.4)	997 (28.1)	−4.3%	−2.7%
60-d MDRO infection (respiratory), n (%)	148 (4.2)	54 (5.5)	1.3%	169 (4.6)	187 (5.3)	0.7%	0.6%
60-d MDRO infection (any site), n (%)	164 (4.6)	60 (6.2)	1.6%	180 (4.9)	189 (5.3)	0.4%	1.2%
*C. difficile* infection, n (%)^b^	123 (3.5)	24 (2.5)	−1.0%	141 (3.8)	120 (3.4)	−0.4%	−0.6%
Respiratory culture-positive for a BIOFIRE^®^ PN Panel bacterial pathogen (N = 3374)							
Early appropriate therapy within 0-24 h, n (%)^c^	659 (67.0)	231 (74.8)	7.8%	761 (71.1)	725 (71.7)	0.6%	7.2%
Early antimicrobial de-escalation within 24–48 h, n (%)^a^	327 (33.3)	122 (39.5)	6.2%	345 (32.2)	326 (32.2)	0.0%	6.2%
ICU mortality, n (%)	125 (12.7)	58 (18.8)	6.1%	115 (10.7)	200 (19.8)	9.1%	−3.0%
30-d all-cause mortality, n (%)	272 (27.2)	111 (35.9)	8.7%	281 (26.2)	338 (33.4)	7.2%	1.5%
90-d readmission, n (%)	360 (36.6)	80 (25.9)	−10.7%	379 (35.4)	286 (28.3)	−7.1%	−3.6%
60-d MDRO infection (respiratory), n (%)	74 (7.5)	26 (8.4)	0.9%	95 (8.9)	103 (10.2)	1.3%	−0.4%
60-d MDRO infection (any site), n (%)	82 (8.3)	30 (9.7)	1.4%	100 (9.3)	104 (10.3)	1.0%	0.4%
*C. difficile* infection, n (%)^b^	39 (4.0)	11 (3.6)	−0.4%	57 (5.3)	51 (5.0)	−0.3%	−0.1%

Abbreviation: MDRO, multidrug-resistant organism.

^a^De-escalation determined based on lowering of total daily antibiotic spectrum scores within 24–48 h interval.

^b^Within 90 d of index pneumonia episode.

^c^Early appropriate therapy was defined as administration of at least 1 dose of an antimicrobial agent to which the corresponding organism was susceptible or inferred to be susceptible based on culture results; for polymicrobial cultures, appropriateness required coverage of all organisms.

Preintervention trends in early antimicrobial de-escalation and early appropriate therapy within 24 hours were visually similar between groups (Supplementary Figure 4). In weighted regression models restricted to the preintervention period, interaction terms between event time and treatment group were not statistically significant for early antimicrobial de-escalation (*P* = .489) or early appropriate therapy (*P* = .500), consistent with the parallel trends assumption.

Among culture-positive patients, early appropriate therapy within 0–24 hours significantly improved following PN Panel implementation in both unadjusted (pre–post difference, 7.8% in PN Panel hospitals vs 0.6% in control hospitals; DiD, 7.2%; RR, 1.09; 95% CI, 1.02–1.16; *P* = .017) and adjusted analyses (aRR, 1.11; 95% CI, 1.04–1.18; *P* = .001; Figure 1 and Supplementary Table 5). PN Panel implementation was also associated with a significant increase in early antimicrobial de-escalation among culture-positive patients after IPTW adjustment (aRR, 1.23; 95% CI, 1.02–1.50; *P* = .032).The proportion of patients receiving early appropriate therapy was similar regardless of ASC de-escalation status (23.9% without de-escalation vs 22.8% with de-escalation), and there was no statistically significant association between ASC de-escalation and early appropriate therapy (*P* = .18).

Secondary outcomes (ICU mortality, 30-day all-cause mortality, 90-day hospital readmission, 60-day incidence of MDR infection, and 90-day incidence of CDI) were generally similar between groups; however, ICU mortality was significantly lower following PN Panel implementation among culture-positive patients (aRR, 0.70; 95% CI, 0.50–0.97; *P* = .035; Figure 1 and Supplementary Table 5).

### ASP Subgroup Analyses

The effect of PN Panel implementation on early antimicrobial de-escalation, early appropriate therapy, and mortality by hospital ASP practices is shown in Table 4 and Supplementary Tables 6–8. No significant effect modification of PN Panel implementation on early antimicrobial de-escalation was observed across ASP practices, except for hospitals with automatic antibiotic stop orders (IPTW-adjusted 3-way interaction *P* = .043). In this subgroup (n = 10 011), PN Panel implementation significantly improved early antimicrobial de-escalation (aRR, 1.21; 95% CI, 1.09–1.35; *P* < .001). In contrast, no significant effect was observed among patients treated in hospitals without automatic antibiotic stop orders (n = 1735; aRR, 0.88; 95% CI, 0.66–1.17; *P* = .398).

**Table 4.** Difference-in-Differences IPTW-Adjusted Poisson Regression Analyses of Early Antimicrobial De-Escalation Within 24–48 h, Early Appropriate Therapy Within 0–24 h, and 30-d Mortality Following PN Panel Implementation, Stratified by Antimicrobial Stewardship Characteristics

	Early Antimicrobial De-Escalation Within 24–48 h			Early Appropriate Therapy Within 0–24 h^a^			30-d Mortality		
Stratum	Risk Ratio (95% CI)	*P*	3-way Interaction *P* Value	Risk Ratio (95% CI)	*P*	3-way Interaction *P* Value	Risk Ratio (95% CI)	*P*	3-way Interaction *P* Value
Presence of an infectious diseases consult service									
Yes (n = 11,583)	1.16 (1.05–1.28)	.004	.961	0.89 (0.73–1.11)	.321	.063	0.97 (0.86–1.09)	.619	.031
No (n = 163)	1.13 (0.45–2.87)	.794		9.41 (0.91–98.21)	.068		3.41 (1.13–10.8)	.032	
Automatic ID consults (any)									
Yes (n = 7,020)	1.10 (0.96–1.25)	.169	.252	0.89 (0.67–1.18)	.428	.656	0.93 (0.78–1.08)	.318	.141
No (n = 4,726)	1.24 (1.05–1.47)	.011		0.99 (0.71–1.37)	.927		1.10 (0.92–1.34)	.286	
Presence of an ID-trained pharmacist									
Yes (n = 10,662)	1.16 (1.04–1.29)	.008	…	1.01 (0.81–1.26)	.916	…	1.04 (0.93–1.18)	.493	…
No (n = 1,084)^b^	…	…		…	…		…	…	
ASP weekly review: ≥3 d vs 0–2 d									
Yes (n = 10,721)	1.15 (1.02–1.28)	.007		1.18 (1.02–1.35)	.019		0.98 (0.86–1.10)	.696	
No (n = 1,0,25)	…	…		…	…		…	…	
Presence of an antibiotic timeout policy									
Yes (n = 5,605)	1.10 (0.94–1.27)	.257	.280	0.86 (0.63–1.15)	.300	.577	0.95 (0.80–1.13)	.560	.639
No (n = 6,141)	1.22 (1.07–1.40)	.003		0.96 (0.72–1.30)	.801		1.01 (0.86–1.18)	.950	
Automatic antibiotic stop orders									
Yes (n = 10,011)	1.21 (1.09–1.35)	<.001	.043	1.01 (0.89–1.27)	.937	.028	1.04 (0.93–1.19)	.464	.015
No (n = 1,735)	0.88 (0.66–1.17)	.398		0.51 (0.28–0.89)	.021		0.68 (0.49–0.94)	.022	
MRSA nares protocol									
Yes (n = 5,205)	1.21 (1.04–1.42)	.015	.382	1.03 (0.94–1.13)	.531	.032	1.12 (0.94–1.35)	.194	.048
No (n = 6,541)	1.11 (0.97–1.26)	.129		1.18 (1.08–1.28)	<.001		0.89 (0.77–1.03)	.129	
CAP treatment pathway									
Yes (n = 10,755)	1.17 (1.06–1.31)	.002	.424	1.11 (1.04–1.18)	.002	.518	1.01 (0.90–1.14)	.850	.546
No (n = 991)	0.99 (0.66–1.54)	.986		1.02 (0.79–1.31)	.884		1.19 (0.71–2.10)	.534	
HAP treatment pathway									
Yes (n = 8,508)	1.15 (1.03–1.30)	.017	.984	1.11 (1.03–1.19)	.005	.433	0.99 (0.87–1.13)	.913	.307
No (n = 3,238)	1.15 (0.93–1.43)	.210		1.05 (0.91–1.20)	.531		1.15 (0.88–1.52)	.303	
Sepsis treatment pathway									
Yes (n = 8,900)	1.12 (1.01–1.26)	.043	.448	1.13 (1.05–1.21)	<.001	.168	0.95 (0.83–1.08)	.049	.009
No (n = 2,846)	0.99 (0.70–1.42)	.936		0.97 (0.76–1.24)	.780		1.71 (1.10–2.78)	.021	
Vancomycin de-escalation protocol									
Yes (n = 5,205)	1.21 (1.04–1.42)	.015	.382	1.11 (0.79–1.56)	.545	.154	1.13 (0.94–1.35)	.194	.049
No (n = 6,541)	1.11 (0.97–1.27)	.129		0.82 (0.62–1.07)	.128		0.89 (0.77–1.03)	.129	

A risk ratio >1 indicates higher rates of the outcome (early antimicrobial de-escalation within 24-48 h, early appropriate therapy within 0-24 h, 30-d mortality) associated with BIOFIRE^®^ PN Panel implementation (post- vs preimplementation, relative to controls).

Abbreviations: ASP, antimicrobial stewardship program; CI, confidence interval.

^a^Only includes bacterial culture-positive patients as appropriateness cannot be assessed in culture-negative patients.

^b^All patients in the “No” subgroup were from control hospitals; no BIOFIRE^®^ PN Panel sites were represented in this subset.

For early appropriate therapy, significant effect modification was observed for the presence of automatic antibiotic stop orders (IPTW-adjusted 3-way interaction *P* = .028) and MRSA nares screening protocols (IPTW-adjusted 3-way interaction *P* = .032). Among patients treated at hospitals without automatic antibiotic stop orders (n = 553), PN Panel implementation was associated with a lower likelihood of early appropriate therapy (aRR, 0.51; 95% CI, 0.28–0.89; *P* = .021), whereas no significant effect was observed at hospitals with automatic antibiotic stop orders (aRR, 1.01; 95% CI, 0.89–1.27; *P* = .937). Conversely, PN Panel implementation significantly improved early appropriate therapy in hospitals without an MRSA nares screening protocol (aRR, 1.18; 95% CI, 1.08–1.28; *P* < .001), while no improvement was observed in hospitals with an established MRSA nares protocol (aRR, 1.03; 95% CI, 0.94–1.13; *P* = .531).

Regarding 30-day mortality, significant interaction was observed for ID consult service (*P* = .031), automatic antibiotic stop orders (*P* = .015), MRSA nares protocol (*P* = .048), sepsis treatment pathway (*P* = .009), and vancomycin de-escalation protocol (*P* = .049). Notably, lack of ID consult services was associated with a significant increase in 30-day mortality (aRR, 3.41; 95% CI, 1.13–10.8; *P* = .032), as well as lack of sepsis treatment protocol (aRR, 1.71; 95% CI, 1.10–2.78; *P* = .021).

### Outcomes Stratified by Pneumonia Type

Stratified analyses by pneumonia type showed similar overall patterns (Supplementary Table 9). Among patients with hospital-acquired pneumonia (HAP), PN Panel implementation was not associated with early antimicrobial de-escalation (aRR, 1.12; 95% CI, 0.95–1.32; *P* = .148), but was associated with higher early appropriate therapy (aRR, 1.14; 95% CI, 1.04–1.25; *P* = .006), with a nonsignificant reduction in 30-day mortality (aRR, 0.88; 95% CI, 0.76–1.02; *P* = .083). Among patients without HAP, PN Panel implementation was associated with increased early antimicrobial de-escalation (aRR, 1.20; 95% CI, 1.05–1.37; *P* = .007), with no significant differences in early appropriate therapy or 30-day mortality. There was no evidence of effect modification by pneumonia type for early antimicrobial de-escalation or early appropriate therapy (interaction *P* = .526 and *P* = .280), although a significant interaction was observed for 30-day mortality (*P* = .035).

## DISCUSSION

In this national cohort of VHA ICU patients with pneumonia, BIOFIRE® PN Panel implementation was associated with improved antimicrobial optimization, including higher likelihood of appropriate therapy within 24 hours and earlier de-escalation to narrower-spectrum regimens. PN Panel implementation was also associated with lower ICU mortality among culture-positive patients, suggesting potential short-term clinical benefit during the acute phase of illness, although 30-day all-cause mortality did not differ overall. No differences were observed in 90-day readmissions, MDRO infections, or CDI. Together with prior studies [4–8, 21, 26–29], these findings provide real-world evidence that RDTs can meaningfully influence antimicrobial prescribing in critically ill pneumonia patients.

A unique feature of this study was the ability to evaluate PN Panel implementation across a national cohort of VA hospitals while examining heterogeneity in outcomes by ASP infrastructure [19]. Improvements were more pronounced in hospitals with structured stewardship activities, such as standardized reassessment policies and treatment pathways (eg, sepsis), while higher mortality in hospitals lacking ID consult services highlights the importance of ID specialist engagement in maximizing the clinical impact of RDTs. The presence of automatic stop orders was associated with higher rates of early antimicrobial de-escalation and neutral effects on early appropriate therapy and 30-day mortality, whereas absence of stop orders was paradoxically associated with higher de-escalation and lower 30-day mortality. These findings likely reflect differences in how stop orders are operationalized, including timing of review, adherence to renewal prompts, and selective application to specific antimicrobials. The independent influence of ASP pharmacist involvement and frequency of daily ASP reviews could not be assessed because all PN Panel sites had dedicated ASP pharmacist involvement and conducted ASP reviews at least 3 times weekly.

While the ASP subgroup findings suggest an interaction between RDTs and stewardship infrastructure, these associations should be interpreted cautiously. Limitations include smaller sample sizes in some subgroups, particularly hospitals without automatic antibiotic stop orders, the risk of type I error from multiple comparisons, and the ecological nature of ASP variables measured at the hospital level. ASP characteristics were derived from the 2020 VHA HAIG ASP Survey and reflect reported practices rather than operational implementation, with limited detail on antimicrobial scope, duration thresholds, or adherence to renewal prompts [19]. Exposure to MRSA screening protocols were similarly measured at the hospital level based on responses to conditional survey questions, and patient-level data were unavailable. Consequently, variability in stewardship implementation and other unmeasured hospital-level factors may have influenced observed associations.

This study has several notable strengths. Use of a matched quasi-experimental DiD design accounted for the temporal overlap between PN Panel adoption and the COVID-19 pandemic, a period marked by major changes in healthcare delivery, antimicrobial use, and patient mix. Incorporation of contemporaneous control hospitals minimized bias from secular trends and strengthened the validity of these findings [13, 14], and IPTW further balanced baseline differences between groups. The national VHA network also enhances generalizability across diverse practice environments with varying ASP and ID capacity [22].

Several limitations should also be considered. Residual or unmeasured confounding cannot be excluded given the retrospective observational design. The postimplementation cohort included only patients whose clinicians ordered the PN Panel on the same respiratory specimen as the index culture, reflecting real-world use but introducing potential selection bias. ASP characteristics were measured at the facility level and may not reflect patient-level exposure to stewardship interventions. The analysis was limited to ICU patients, and generalizability to ward or outpatient populations is uncertain. Information on other viral diagnostics (eg, influenza or RSV multiplex testing) and additional tests, including urinary antigen assays, beyond SARS-CoV-2 was unavailable; however, these practices would be expected to influence intervention and control hospitals similarly given the DiD design. Differences in how RDT results were incorporated into local workflows may also have influenced observed effects. Finally, antimicrobial resistance rates were relatively low, and future studies should evaluate PN Panel implementation in higher-resistance settings.

Despite these limitations, the BIOFIRE® PN Panel demonstrated strong analytic performance, including high PPA/NPA, and complements prior studies [4–8, 21, 26–29]. To our knowledge, this represents the largest multicenter evaluation of PN Panel implementation to date, providing real-world evidence of its clinical impact beyond controlled microbiology trial settings. The integrated VHA system, with standardized data infrastructure and heterogeneous stewardship environments, provides a natural experiment to evaluate how RDTs influence antimicrobial decision-making in routine practice.

In conclusion, implementation of the BIOFIRE® PN Panel was associated with earlier appropriate therapy and more timely de-escalation of broad-spectrum antimicrobials in a national cohort of critically ill pneumonia patients. The DiD design with contemporaneous controls strengthened the validity of these findings and accounted for secular changes during the COVID-19 pandemic. Hospitals with well-developed ASP programs and structured stewardship activities showed the most consistent improvements in antimicrobial optimization, underscoring the importance of integrating RDT platforms within robust ASP and ID frameworks.

## Supplementary Material

ciag328_Supplementary_Data

## Supplementary Data

Supplementary materials are available at *Clinical Infectious Diseases* online. Consisting of data provided by the authors to benefit the reader, the posted materials are not copyedited and are the sole responsibility of the authors, so questions or comments should be addressed to the corresponding author.
