# Supplementary material for: Clinical Impact of the BIOFIRE® FILMARRAY® Pneumonia Panel in the Veterans Affairs Healthcare System: A Difference-in-Differences Study
Source: Clin Infect Dis. 2026 Jun 12;83(3):e461–71. doi: 10.1093/cid/ciag328 (PMC13644875; doi:10.1093/cid/ciag328)
Supplement: Supplementary file 1 [file ciag328_supplementary_data.pdf]

**Supplemental Figure 1.** Study Attrition

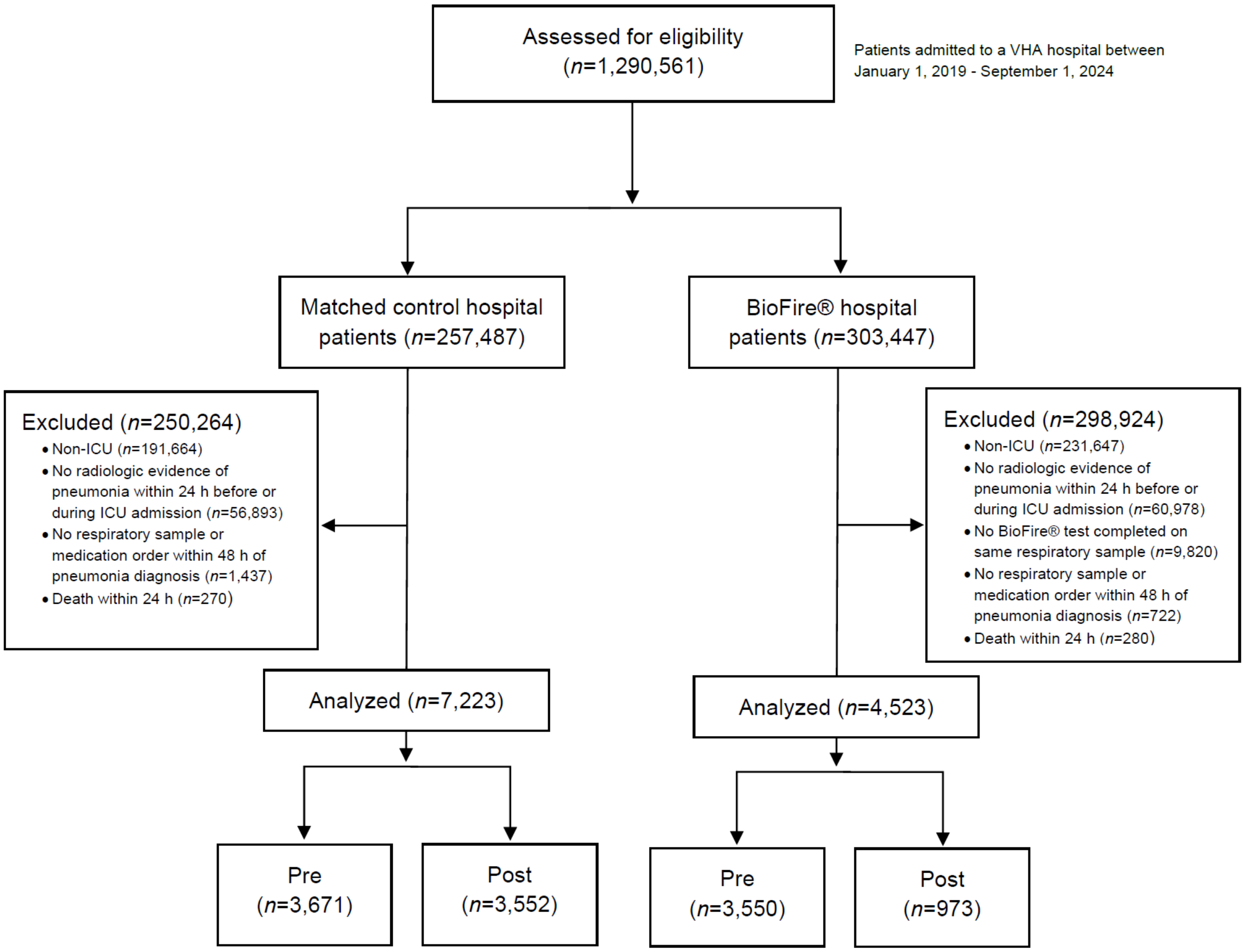


**Footnote:** Flow diagram of patient selection and cohort derivation for PN Panel and matched control hospitals. Patients were identified from VHA hospital admissions and sequentially excluded based on prespecified criteria, including absence of ICU admission, lack of radiologic evidence of pneumonia, absence of a respiratory specimen or antimicrobial therapy within the defined time window, and early death. Final analytic cohorts were stratified by pre- and post-implementation periods relative to PN Panel adoption.

**Supplemental Figure 2.** Covariate Balance Before and After Inverse Probability of Treatment Weighting (IPTW)

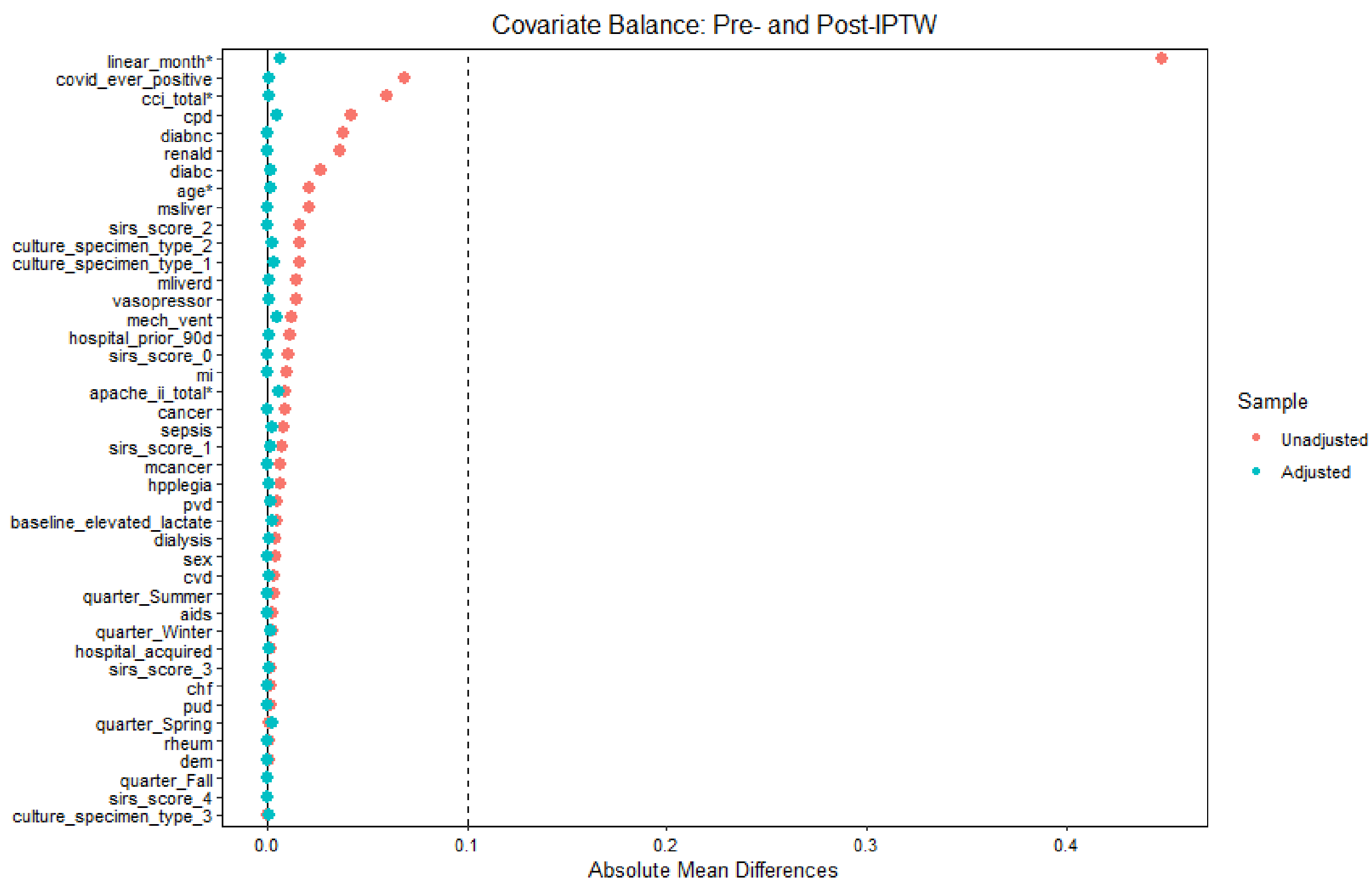


**Footnote:** Absolute standardized mean differences for baseline covariates comparing PN Panel exposure groups before (unadjusted) and after IPTW adjustment. Each point represents a covariate, with values closer to zero indicating improved balance. The dashed vertical line at 0.1 denotes the commonly accepted threshold for adequate covariate balance.

**Supplemental Figure 3.** Distribution of Antibiotic Spectrum Coverage (ASC) Scores at 24–48 Hours Post-Implementation

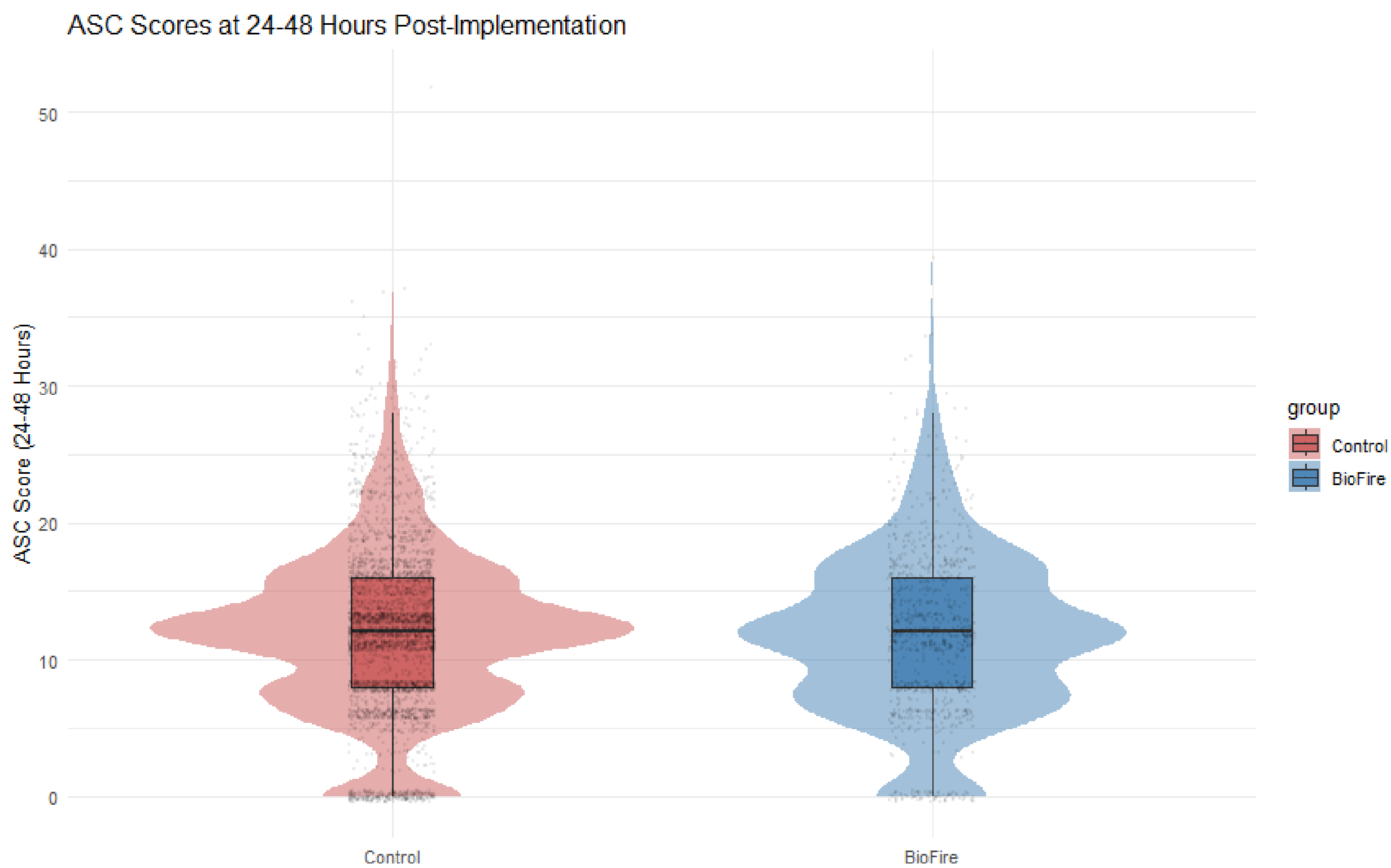


**Footnote:** Violin plots with overlaid boxplots showing the distribution of ASC scores at 24–48 hours of antimicrobial therapy among patients in PN Panel and control hospitals during the post-implementation period. Median ASC scores were similar between groups (PN Panel, 12 [IQR, 8–16] vs control, 12 [IQR, 8–16]; P=0.154). Points represent individual patient observations.

**Supplemental Figure 4.** Pre-Intervention Trends in Early Antimicrobial De-Escalation and Early Appropriate Therapy

**A. Early antimicrobial de-escalation within 24–48 hours in the pre-intervention period**

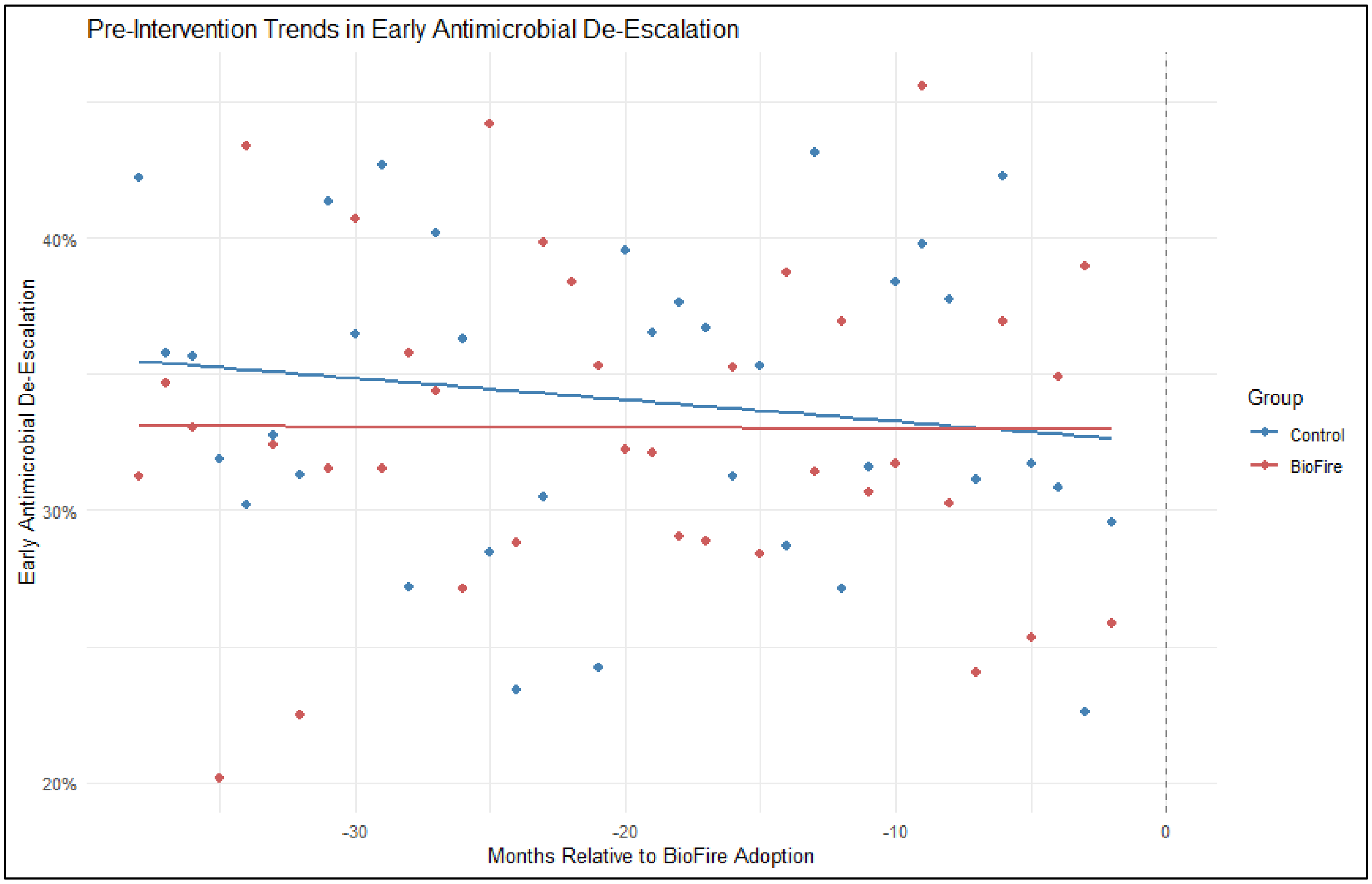

**B. Early appropriate therapy within 24 hours in the pre-intervention period**

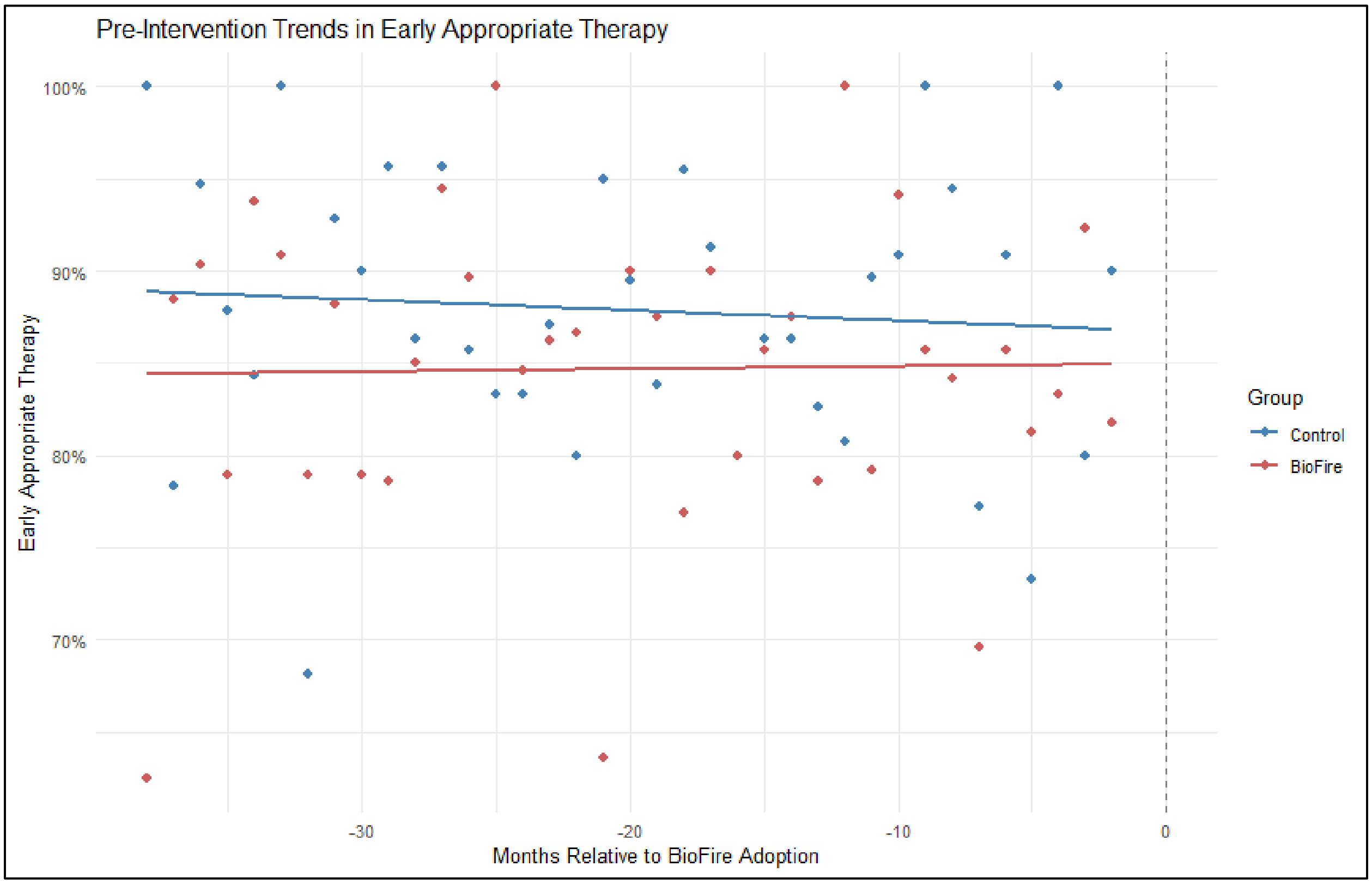


**Footnote:** (A) Early antimicrobial de-escalation within 24–48 hours and (B) early appropriate therapy within 24 hours in the pre-intervention period, plotted by months relative to PN Panel implementation. Points represent monthly estimates, and lines represent fitted linear trends for intervention (BIOFIRE® PN Panel) and matched control hospitals. The vertical dashed line indicates the time of PN Panel implementation (time 0). Pre-intervention trends were visually similar between groups, supporting the parallel trends assumption underlying the difference-in-differences analysis.

**Supplemental Table 1.** Concordance of PN Panel and Respiratory Culture Results for BIOFIRE-Detected Bacterial Pathogens in PN Panel Hospitals, Post-Implementation[a]

| Target | PN+ CX+ | PN+ CX- | PN- CX+ | PN- CX- | PPA [b] | NPA [b] |
|---|---|---|---|---|---|---|
| *Staphylococcus aureus* | 111 | 100 | 1 | 758 | 99.1% | 88.3% |
| *Streptococcus agalactiae* | 3 | 41 | 0 | 927 | 100% | 95.7% |
| *Streptococcus pneumoniae* | 26 | 39 | 0 | 906 | 100% | 95.9% |
| *Streptococcus pyogenes* | 0 | 4 | 0 | 969 | NA | 100% |
| *Proteus spp.* | 21 | 17 | 2 | 931 | 91.3% | 98.2% |
| *Acinetobacter baumannii* complex | 4 | 8 | 1 | 958 | 80.0% | 99.2% |
| *Enterobacter cloacae* complex | 12 | 20 | 3 | 936 | 80.0% | 97.9% |
| *Escherichia coli* | 26 | 38 | 3 | 904 | 89.7% | 96.0% |
| *Haemophilus influenzae* | 24 | 87 | 0 | 859 | 100% | 90.8% |
| *Klebsiella aerogenes* | 13 | 6 | 2 | 950 | 86.7% | 99.4% |
| *Klebsiella oxytoca* | 8 | 13 | 1 | 949 | 88.9% | 98.7% |
| *Klebsiella pneumoniae* group | 37 | 40 | 1 | 891 | 97.4% | 95.7% |
| *Moraxella catarrhalis* | 10 | 23 | 0 | 925 | 100% | 97.8% |

| | | | | | | |
|---|---|---|---|---|---|---|
| *Pseudomonas aeruginosa* | 88 | 36 | 3 | 844 | 96.7% | 95.9% |
| *Serratia marcescens* | 10 | 21 | 3 | 937 | 76.9% | 97.8% |
| **Grand Total** | **393** | **493** | **20** | **13644** | **95.2%** | **96.5%** |

[a]For sites reporting partial results from the Panel, only reported results were included in calculations. This included n=2 where on resistance gene targets were reported.

[b]Reference group: standard culture

Abbreviations: PN+, BIOFIRE(R) Pneumonia Panel positive; CX +, culture positive; PN-, BIOFIRE(R) Pneumonia Panel; CX-, culture negative; PPA, positive percent agreement; NPA, negative percent agreement.

**Supplemental Table 2**. Resistance Genes Identified by the PN Panel in the Post-Implementation Group in PN Panel Hospitals

| Resistance Gene | Post-PN(n=973) |
|---|---|
| Gram-positive resistance gene detected, *n* (%) | |
| mecA/C/MREJ* | 86/681 (8.8) |
| Gram-negative resistance gene detected, *n* (%) | |
| CTX-M | 26/688 (2.7) |
| IMP | 1/688 (0.1) |
| VIM | 0/528 (0.0) |
| NDM | 0/688 (0.0) |
| KPC | 1/685 (0.1) |
| OXA-48-like | 1/675 (0.1) |

[a] For sites reporting partial results from the Panel, only reported results were included in calculations

**Supplemental Table 3.** Atypical Pathogens and Viruses Identified by PN Panel in the Post-Implementation Group in PN Panel Hospitals

| Pathogen | Post-PN (n=973) |
|---|---|
| Atypical organism, *n* (%) | 5 (0.5) |
| *Chlamydia pneumoniae* | 0 (0) |
| *Legionella pneumophilia* | 5 (0.5) |
| *Mycoplasma pneumoniae* | 0 (0) |
| Respiratory virus, *n* (%) | 113 (11.6) |
| Human metapneumovirus | 11 (1.1) |
| Parainfluenza virus | 18 (1.8) |
| Influenza A virus | 13 (1.3) |
| Influenza B virus | 0 (0) |
| Coronavirus | 28 (2.9) |
| Rhinovirus/enterovirus | 36 (3.7) |
| Respiratory syncytial virus | 10 (1.0) |
| Adenovirus | 2 (0.2) |

Co-infections, limited to bacterial targets identified by the PN Panel, were detected in 71 patients (7.3%), including 3 bacterial/atypical and 68 bacterial/viral combinations.

**Supplemental Table 4.** Non-Panel Bacterial Pathogens Isolated from Respiratory Cultures in the Post-Implementation Group in PN Panel Hospitals

| | | | |
|---|---|---|---|
| *Achromobacter xylosoxidans* | 1 | *Haemophilus parainfluenzae* | 5 |
| *Aggregatibacter aphrophilus* | 1 | *Hafnia alvei* | 2 |
| *Alcaligenes faecalis* | 1 | *Streptococcus spp. (non-Panel or unspeciated)* | 16 |
| *Bordetella bronchiseptica* | 1 | *Morganella morganii* | 1 |
| *Burkholderia spp.* | 1 | *Neisseria spp.* | 1 |
| *Citrobacter spp.* | 3 | *Pastuerella multocida* | 1 |
| *Corynebacterium spp.* | 7 | *Providencia stuartii* | 2 |
| *Enterobacter spp. (non-Panel or unspeciated)* | 1 | *Pseudomonas spp. (non-Panel or unspeciated)* | 5 |
| *Enterococcus faecium* | 1 | *Rothia mucilaginosa* | 2 |
| *Gram-negative rod (unspeciated)* | 2 | *Sphingomonas paucimobilis* | 1 |
| *Gram-positive rod (unspeciated)* | 1 | *Stenotrophomonas maltophilia* | 14 |
| *Haemophilus parahaemolyticus* | 1 | *Coagulase- negative Staphylococcus spp.* | 5 |

**Supplemental Table 5**. Difference-in-Differences Poisson Regression Analysis of Outcomes Associated with PN Panel Implementation

| | **Unadjusted** | | **IPTW-Adjusted** | |
|---|---|---|---|---|
| Outcome | Risk Ratio (95% CI) | *P* | Risk Ratio (95% CI) | *P* |
| **Overall (*N*=11,746)** | | | | |
| Early antimicrobial de-escalation within 24-48 hours[a] | 1.13 (1.01-1.27) | 0.027 | 1.14 (1.04-1.26) | 0.007 |
| ICU mortality | 1.02 (0.85-1.23) | 0.846 | 0.92 (0.77-1.09) | 0.318 |
| 30-day all-cause mortality | 1.08 (0.95-1.22) | 0.239 | 0.99 (0.89-1.12) | 0.797 |
| 90-day readmission | 0.89 (0.77-1.01) | 0.063 | 0.94 (0.84-1.06) | 0.328 |
| 60-day MDRO infection (respiratory) | 1.16 (0.80-1.67) | 0.416 | 1.22 (0.87-1.70) | 0.230 |
| 60-day MDRO infection (any site) | 1.23 (0.86-1.74) | 0.247 | 1.32 (0.96-1.81) | 0.088 |
| *Clostridioides difficile* infection [c] | 0.81 (0.49-1.31) | 0.399 | 0.90 (0.59-1.37) | 0.619 |
| **Respiratory Culture-Positive for a PN Panel Bacterial Pathogen (N=3,374)** | | | | |
| Early appropriate therapy within 24 hours[c] | 1.09 (1.02-1.16) | 0.017 | 1.11 (1.04-1.18) | 0.001 |
| Early antimicrobial de-escalation within 24-48 hours[a] | 1.19 (0.96-1.46) | 0.113 | 1.23 (1.02-1.50) | 0.032 |
| ICU mortality | 0.80 (0.55-1.14) | 0.224 | 0.70 (0.50-0.97) | 0.035 |
| 30-day all-cause mortality | 1.02 (0.81-1.28) | 0.873 | 0.91 (0.74-1.13) | 0.395 |
| 90-day readmission | 0.88 (0.70-1.12) | 0.306 | 0.96 (0.78-1.18) | 0.720 |
| 60-day MDRO infection (respiratory) | 0.97 (0.58-1.59) | 0.916 | 1.07 (0.68-1.69) | 0.759 |
| 60-day MDRO infection (any site) | 1.05 (0.65-1.69) | 0.821 | 1.20 (0.78-1.85) | 0.408 |
| *C. difficile* infection [b] | 0.95 (0.43-1.96) | 0.886 | 0.90 (0.58-1.37) | 0.619 |

[a]De-escalation determined based on lowering of total daily antibiotic spectrum scores within 24-48 hours

[b]Within 90 days of index pneumonia episode

[c]Early appropriate therapy was defined as administration of at least one dose of an antimicrobial agent to which the corresponding organism was susceptible or inferred to be susceptible based on culture results; for polymicrobial cultures, appropriateness required coverage of all organisms.

**Supplemental Table 6.** Difference-in-Differences Poisson Regression Analyses of Early Antimicrobial De-Escalation Following PN Panel Implementation, Stratified by Antimicrobial Stewardship Characteristics

| | **Unadjusted** | | | **IPTW-Adjusted** | | |
|---|---|---|---|---|---|---|
| Stratum | Risk Ratio (95% CI) | *P* | 3-way Interaction P-value | Risk Ratio (95% CI) | *P* | 3-way Interaction *P*-value |
| **Presence of an Infectious Diseases Consult Service** | | | 0.954 | | | 0.961 |
| Yes (*n*=11,583) | 1.13 (1.01-1.27) | 0.030 | | 1.16 (1.05-1.28) | 0.004 | |
| No (*n*=169) | 1.10 (0.42-2.92) | 0.844 | | 1.13 (0.45-2.87) | 0.794 | |
| **Automatic ID Consults (Any)** | | | 0.223 | | | 0.252 |
| Yes (*n*=7,020) | 1.06 (0.91-1.24) | 0.441 | | 1.10 (0.96-1.25) | 0.169 | |
| No (*n*=4,726) | 1.23 (1.04-1.46) | 0.018 | | 1.24 (1.05-1.47) | 0.011 | |
| **Presence of an ID-Trained Pharmacist** | | | --- | | | --- |
| Yes (*n*=10,662) | 1.14 (1.01-1.28) | 0.032 | | 1.16 (1.04-1.29) | 0.008 | |
| No (*n*=1,084)[a] | --- | --- | | --- | --- | |
| **ASP weekly review: ≥ 3 days vs 0-2 days** | | | --- | | | --- |
| Yes (*n*=1,025) | 1.13 (1.01-1.38) | 0.034 | | 1.15 (1.02-1.28) | 0.007 | |
| No (*n*=10,721) | --- | --- | | --- | --- | |

| | | | | | | |
|---|---|---|---|---|---|---|
| **Presence of an Antibiotic Timeout Policy** | | | 0.234 | | | 0.280 |
| Yes (*n*=5,605) | 1.05 (0.89-1.24) | 0.557 | | 1.10 (0.94-1.27) | 0.257 | |
| No (*n*=6,141) | 1.21 (1.03-1.41) | 0.017 | | 1.22 (1.07-1.40) | 0.003 | |
| **Automatic Antibiotic Stop Orders** | | | 0.118 | | | **0.043** |
| Yes (*n*=10,011) | 1.18 (1.05-1.33) | 0.006 | | 1.21 (1.09-1.35) | <0.001 | |
| No (*n*=1,735) | 0.87 (0.61-1.23) | 0.452 | | 0.88 (0.66-1.17) | 0.398 | |
| **MRSA Nares Protocol** | | | 0.648 | | | 0.382 |
| Yes (*n*=5,205) | 1.16 (0.97-1.38) | 0.101 | | 1.21 (1.04-1.42) | 0.015 | |
| No (*n*=6,541) | 1.10 (0.95-1.27) | 0.206 | | 1.11 (0.97-1.26) | 0.129 | |
| **CAP Treatment Pathway** | | | 0.374 | | | 0.424 |
| Yes (*n*=10,775) | 1.15 (1.02-1.30) | 0.021 | | 1.17 (1.06-1.31 | 0.002 | |
| No (*n*=991) | 0.96 (0.65-1.41) | 0.834 | | 0.99 (0.66-1.54) | 0.986 | |
| **HAP Treatment Pathway** | | | 0.815 | | | 0.984 |
| Yes (*n*=8,508) | 1.14 (0.99-1.31) | 0.058 | | 1.15 (1.03-1.30) | 0.017 | |
| No (*n*=3,238 | 1.10 (0.89-1.37) | 0.374 | | 1.15 (0.93-1.43) | 0.210 | |

| | | | | | | |
|---|---|---|---|---|---|---|
| **Sepsis Treatment Pathway** | | | 0.377 | | | 0.448 |
| Yes (*n*=8,900) | 1.10 (0.97-1.27) | 0.128 | | 1.12 (1.01-1.26) | 0.043 | |
| No (*n*=2,846) | 0.95 (0.71-1.30) | 0.777 | | 0.99 (0.70-1.42) | 0.936 | |
| **Vancomycin De-Escalation Protocol** | | | 0.648 | | | 0.382 |
| Yes (*n*=5,205) | 1.16 (0.97-1.38) | 0.101 | | 1.21 (1.04-1.42) | 0.015 | |
| No (*n*=6,541) | 1.10 (0.95) | 0.206 | | 1.11 (0.97-1.27) | 0.129 | |

Abbreviations: CI, confidence interval; ASP, antimicrobial stewardship program

For early antimicrobial de-escalation within 24 hours and de-escalation within 24-48 hours, a risk ratio > 1 indicates higher rates of early antimicrobial de-escalation within 24 hours and de-escalation within 24-48 hours associated with BIOFIRE® PN Panel implementation.

For 30-day mortality, a risk ratio > 1 indicates higher rates of 30-day mortality with BIOFIRE® PN Panel implementation.

[a]All patients in the "No" subgroup were from control hospitals; no BIOFIRE® PN Panel sites were represented in this subset.

**Supplemental Table 7.** Difference-in-Differences Poisson Regression Analyses of Early Appropriate Therapy within 24 hours Among Bacterial Culture-Positive Patients Following Pneumonia Panel Implementation, Stratified by Antimicrobial Stewardship Characteristics

| | Unadjusted | | | IPTW-Adjusted | | |
|---|---|---|---|---|---|---|
| Stratum | Risk Ratio (95% CI) | *P* | 3-way Interaction P-value | Risk Ratio (95% CI) | *P* | 3-way Interaction P-value |
| **Presence of an Infectious Diseases Consult Service** | | | 0.049 | | | 0.063 |
| Yes (*n*=3,331) | 1.00 (0.79-1.26) | 0.999 | | 0.89 (0.73-1.11) | 0.321 | |
| No (*n*=43) | 9.64 (0.96-97.1) | 0.062 | | 9.41 (0.91-98.21) | 0.068 | |
| **Automatic ID Consults (Any)** | | | 0.681 | | | 0.656 |
| Yes (*n*=2,048) | 0.99 (0.72-1.35) | 0.954 | | 0.89 (0.67-1.18) | 0.428 | |
| No (*n*=1,326) | 1.09 (0.78-1.53) | 0.611 | | 0.99 (0.71-1.37) | 0.927 | |
| **Presence of an ID-Trained Pharmacist** | | | --- | | | --- |
| Yes (*n*=3,059) | 1.13 (0.89-1.42) | 0.322 | | 1.01 (0.81-1.26) | 0.916 | |
| No (*n*=315)[a] | --- | --- | | --- | --- | |
| **ASP weekly review: ≥ 3 days vs 0-2 days** | | | --- | | | --- |
| Yes (*n*=3,111) | 1.11 (0.95-1.31) | 0.195 | | 1.18 (1.02-1.35) | **0.019** | |
| No (*n*=263) | --- | --- | | --- | --- | |
| **Presence of an Antibiotic Timeout Policy** | | | 0.669 | | | 0.577 |
| Yes (*n*=1,709) | 0.96 (0.70-1.31) | 0.784 | | 0.86 (0.63-1.15) | 0.300 | |

| | | | | | | |
|---|---|---|---|---|---|---|
| No (*n*=1,665) | 1.06 (0.75-1.48) | 0.742 | | 0.96 (0.72-1.30) | 0.801 | |
| **Automatic Antibiotic Stop Orders** | | | 0.071 | | | **0.028** |
| Yes (*n*=2,821) | 1.11 (0.87-1.42) | 0.389 | | 1.01 (0.89-1.27) | 0.937 | |
| No (*n*=553) | 0.56 (0.27-1.11) | 0.111 | | 0.51 (0.28-0.89) | 0.021 | |
| **MRSA Nares Protocol** | | | 0.094 | | | **0.032** |
| Yes (*n*=1,479) | 1.02 (0.93-1.13) | 0.678 | | 1.03 (0.94-1.13) | 0.531 | |
| No (*n*=1,895) | 1.15 (1.04-1.26) | **0.005** | | 1.18 (1.08-1.28) | **<0.001** | |
| **CAP Treatment Pathway** | | | 0.556 | | | 0.518 |
| Yes (*n*=3,053) | 1.09 (1.01-1.17) | **0.025** | | 1.11 (1.04-1.18) | **0.002** | |
| No (*n*=321) | 1.01 (0.81-1.28) | 0.906 | | 1.02 (0.79-1.31) | 0.884 | |
| **HAP Treatment Pathway** | | | 0.551 | | | 0.433 |
| Yes (*n*=2,434) | 1.08 (0.99-1.18) | 0.056 | | 1.11 (1.03-1.19) | **0.005** | |
| No (*n*=940) | 1.04 (0.91-1.18) | 0.601 | | 1.05 (0.91-1.20) | 0.531 | |
| **Sepsis Treatment Pathway** | | | 0.155 | | | 0.168 |
| Yes (*n*=2,541) | 1.11 (1.02-1.20) | **0.013** | | 1.13 (1.05-1.21) | **<0.001** | |

| | | | | | | |
|---|---|---|---|---|---|---|
| No (*n*=833) | 0.95 (0.79-1.17) | 0.654 | | 0.97 (0.76-1.24) | 0.780 | |
| **Vancomycin De-Escalation Protocol** | | | 0.124 | | | 0.154 |
| Yes (*n*=1,479) | 1.27 (088-1.82) | 0.189 | | 1.11 (0.79-1.56) | 0.545 | |
| No (*n*=1,895) | 0.88 (0.66-1.19) | 0.416 | | 0.82 (0.62-1.07) | 0.128 | |

Abbreviations: CI, confidence interval; ASP, antimicrobial stewardship program

For early antimicrobial de-escalation within 24 hours and de-escalation within 24-48 hours, a risk ratio > 1 indicates higher rates of early antimicrobial de-escalation within 24 hours and de-escalation within 24-48 hours associated with BIOFIRE® PN Panel implementation.

For 30-day mortality, a risk ratio > 1 indicates higher rates of 30-day mortality with BIOFIRE® PN Panel implementation.

[a]All patients in the “No” subgroup were from control hospitals; no BIOFIRE® PN Panel sites were represented in this subset.

**Supplemental Table 8.** Difference-in-Differences Poisson Regression Analyses of 30-Day Mortality Following PN Panel Implementation, Stratified by Antimicrobial Stewardship Characteristics

| | **Unadjusted** | | | **IPTW-Adjusted** | | |
|---|---|---|---|---|---|---|
| Stratum | Risk Ratio (95% CI) | *P* | 3-way Interaction *P* | Risk Ratio (95% CI) | *P* | 3-way Interaction *P* |
| **Presence of an Infectious Diseases Consult Service** | | | 0.033 | | | **0.031** |
| Yes (*n*=11,583) | 1.06 (0.94-1.21) | 0.350 | | 0.97 (0.86-1.09) | 0.619 | |
| No (*n*=169) | 3.60 (1.12-11.1) | 0.028 | | 3.41 (1.13-10.8) | 0.032 | |
| **Automatic ID Consults (Any)** | | | 0.156 | | | 0.141 |
| Yes (*n*=7,020) | 1.01 (0.85-1.20) | 0.906 | | 0.93 (0.78-1.08) | 0.318 | |
| No (*n*=4,726) | 1.22 (1.01-1.47) | 0.451 | | 1.10 (0.92-1.34) | 0.286 | |
| **Presence of an ID-Trained Pharmacist** | | | --- | | | --- |
| Yes (*n*=10,662) | 1.13 (0.99-1.29) | 0.052 | | 1.04 (0.93-1.18) | 0.493 | |
| No (*n*=1,084)[a] | -- | -- | | --- | --- | |
| **ASP weekly review: ≥ 3 days vs 0-2 days** | | | --- | | | --- |
| Yes (*n*=10,721) | 1.07 (0.93-1.22) | 0.337 | | 0.98 (0.86-1.10) | 0.696 | |

| | | | | | | |
|---|---|---|---|---|---|---|
| No (*n*=1,025) | --- | --- | | --- | --- | |
| **Presence of an Antibiotic Timeout Policy** | | | 0.600 | | | 0.639 |
| Yes (*n*=5,605) | 1.03 (0.86-1.23) | 0.749 | | 0.95 (0.80-1.13) | 0.560 | |
| No (*n*=6,141) | 1.10 (0.92-1.32) | 0.288 | | 1.01 (0.86-1.18) | 0.950 | |
| **Automatic Antibiotic Stop Orders** | | | 0.058 | | | **0.015** |
| Yes (*n*=10,011) | 1.13 (0.99-1.29) | 0.074 | | 1.04 (0.93-1.19) | 0.464 | |
| No (*n*=1,735) | 0.75 (0.50-1.11) | 0.160 | | 0.68 (0.49-0.94) | 0.022 | |
| **MRSA Nares Protocol** | | | 0.066 | | | 0.048 |
| Yes (*n*=5,205) | 1.24 (1.01-1.50) | 0.035 | | 1.12 (0.94-1.35) | 0.194 | |
| No (*n*=6,541) | 0.97 (0.83-1.25) | 0.743 | | 0.89 (0.77-1.03) | 0.129 | |
| **CAP Treatment Pathway** | | | 0.474 | | | 0.546 |
| Yes (*n*=10,775) | 1.10 (0.96-1.25) | 0.169 | | 1.01 (0.90-1.14) | 0.850 | |
| No (*n*=991) | 0.97 (0.83-1.15) | 0.743 | | 1.19 (0.71-2.10) | 0.534 | |
| **HAP Treatment Pathway** | | | 0.283 | | | 0.307 |
| Yes (*n*=8,508) | 1.08 (0.93-1.25) | 0.322 | | 0.99 (0.87-1.13) | 0.913 | |

| | | | | | | |
|---|---|---|---|---|---|---|
| No (*n*=3,238 | 1.27 (0.97-1.65) | 0.077 | | 1.15 (0.88-1.52) | 0.303 | |
| **Sepsis Treatment Pathway** | | | **0.003** | | | **0.009** |
| Yes (*n*=8,900) | 1.02 (0.88-1.20) | 0.707 | | 0.95 (0.83-1.08) | **0.049** | |
| No (*n*=2,846) | 1.88 (1.30-2.78) | **0.001** | | 1.71 (1.10-2.78) | **0.021** | |
| **Vancomycin De-Escalation Protocol** | | | 0.066 | | | 0.049 |
| Yes (*n*=5,205) | 1.24 (1.01-1.51) | 0.035 | | 1.13 (0.94-1.35) | 0.194 | |
| No (*n*=6,541) | 0.97 (0.83-1.15) | 0.743 | | 0.89 (0.77-1.03) | 0.129 | |

Abbreviations: CI, confidence interval; ASP, antimicrobial stewardship program

For early antimicrobial de-escalation within 24 hours and de-escalation within 24-48 hours, a risk ratio > 1 indicates higher rates of early antimicrobial de-escalation within 24 hours and de-escalation within 24-48 hours associated with BIOFIRE® PN Panel implementation.

For 30-day mortality, a risk ratio > 1 indicates higher rates of 30-day mortality with BIOFIRE® PN Panel implementation.

[a]All patients in the "No" subgroup were from control hospitals; no BIOFIRE® PN Panel sites were represented in this subset.

**Supplemental Table 9.** Difference-in-Differences IPTW-Adjusted Analyses of Outcomes Stratified by Pneumonia Type (Hospital-Acquired vs Non–Hospital-Acquired)

| | **Early De-Escalation** | | | **Early Appropriate Therapy < 24 hours[a]** | | | **30-Day Mortality** | | |
|---|---|---|---|---|---|---|---|---|---|
| Stratum | Risk Ratio (95% CI) | *P* | 3-way Interaction *P*-value | Risk Ratio (95% CI) | *P* | 3-way Interaction *P*-value | Risk Ratio (95% CI) | *P* | 3-way Interaction *P*-value |
| **Hospital-Acquired Pneumonia** | | | | | | | | | |
| Yes (*n*=5,313) | 1.12 (0.95 - 1.32) | 0.148 | 0.526 | 1.14 (1.04 - 1.25) | 0.006 | 0.280 | 0.88 (0.76 - 1.02) | 0.083 | **0.035** |
| No (*n*=6,433) | 1.20 (1.05 - 1.37) | **0.007** | | 1.06 (0.99 - 1.15) | 0.099 | | 1.14 (0.94 - 1.37) | 0.185 | |

**Footnotes:**

**Abbreviations:** CI, confidence interval; aRR, adjusted risk ratio; HAP, hospital-acquired pneumonia; PN Panel, BIOFIRE® FILMARRAY® Pneumonia Panel; IPTW, inverse probability of treatment weighting.

Risk ratios represent the relative change in outcomes following PN Panel implementation (post- vs pre-implementation) compared with matched control hospitals using a difference-in-differences framework.

For early antimicrobial de-escalation and early appropriate therapy, aRR >1 indicates higher likelihood of the outcome following PN Panel implementation. For 30-day mortality, aRR >1 indicates higher mortality associated with PN Panel implementation.

a Early appropriate therapy was evaluated only among culture-positive patients.

Three-way interaction P-values assess effect modification by pneumonia type.
